# A multi-parametric prognostic model based on clinicopathologic features: vessels encapsulating tumor clusters and hepatic plates predict overall survival in hepatocellular carcinoma patients

**DOI:** 10.1186/s12967-024-05296-3

**Published:** 2024-05-18

**Authors:** Si-Ping Xiong, Chun-Hua Wang, Mei-fang Zhang, Xia Yang, Jing-Ping Yun, Li-Li Liu

**Affiliations:** 1 State Key Laboratory of Oncology in South China, Guangdong Provincial Clinical Research Center for Cancer, Sun Yat-Sen University Cancer Center, Guangzhou, 510060 P.R. China; 2 Department of Pathology, Sun Yat-Sen University Cancer Center, 651# Dong Feng Road East, Guangzhou, 510060 Guangdong China; 3 Department of Pathology, The Eighth Affiliated Hospital of Sun Yat-Sen University, Shenzhen, 518033 China

**Keywords:** Vessels encapsulating tumor clusters, Hepatocellular carcinoma, LASSO regression, Prognostic model

## Abstract

**Background:**

Vessels encapsulating tumor clusters (VETC) is a newly described vascular pattern that is distinct from microvascular invasion (MVI) in patients with hepatocellular carcinoma (HCC). Despite its importance, the current pathological diagnosis report does not include information on VETC and hepatic plates (HP). We aimed to evaluate the prognostic value of integrating VETC and HP (VETC-HP model) in the assessment of HCC.

**Methods:**

A total of 1255 HCC patients who underwent radical surgery were classified into training (879 patients) and validation (376 patients) cohorts. Additionally, 37 patients treated with lenvatinib were studied, included 31 patients in high-risk group and 6 patients in low-risk group. Least absolute shrinkage and selection operator (LASSO) regression analysis was used to establish a prognostic model for the training set. Harrell’s concordance index (C-index), time-dependent receiver operating characteristics curve (tdROC), and decision curve analysis were utilized to evaluate our model's performance by comparing it to traditional tumor node metastasis (TNM) staging for individualized prognosis.

**Results:**

A prognostic model, VETC-HP model, based on risk scores for overall survival (OS) was established. The VETC-HP model demonstrated robust performance, with area under the curve (AUC) values of 0.832 and 0.780 for predicting 3- and 5-year OS in the training cohort, and 0.805 and 0.750 in the validation cohort, respectively. The model showed superior prediction accuracy and discrimination power compared to TNM staging, with C-index values of 0.753 and 0.672 for OS and disease-free survival (DFS) in the training cohort, and 0.728 and 0.615 in the validation cohort, respectively, compared to 0.626 and 0.573 for TNM staging in the training cohort, and 0.629 and 0.511 in the validation cohort. Thus, VETC-HP model had higher C-index than TNM stage system(p < 0.01).Furthermore, in the high-risk group, lenvatinib alone appeared to offer less clinical benefit but better disease-free survival time.

**Conclusions:**

The VETC-HP model enhances DFS and OS prediction in HCC compared to traditional TNM staging systems. This model enables personalized temporal survival estimation, potentially improving clinical decision-making in surveillance management and treatment strategies.

**Supplementary Information:**

The online version contains supplementary material available at 10.1186/s12967-024-05296-3.

## Introduction

Hepatocellular carcinoma (HCC) ranks third in global mortality and sixth in incidence, with China accounting for nearly half of all cases and deaths, as reported by GLOBOCAN 2022 [1–3]. Early-stage HCC typically presents no discernible clinical symptoms, leading to diagnoses at advanced stages. However, advancements in imaging technology have facilitated early-stage HCC detection, allowing more patients to undergo curative resection [4]. Additionally, immune-checkpoint inhibitors, tyrosine kinase inhibitors, and monoclonal antibodies have shown efficacy in treating HCC patients [5]. Despite these advancements, long-term outcomes for patients remain highly variable, presenting a significant clinical management challenge. Current clinical practice primarily relies on stratified prognosis prediction based on tumor burden and cancer-related symptoms [6]. Therefore, the development of a robust prognosis prediction model is imperative.

To date, gross description of specimens, microscopic description, pathological diagnosis, and immunohistochemical examination results are required for the pathological diagnosis of HCC [7, 8]. The gross description of specimens mainly consists of the features of the tumor, distance between the tumor and resection margin, and relationship between the tumor and blood vessels, bile ducts, and liver envelope. The microscopic description comprises the degree and range of tumor necrosis, microvascular invasion (MVI), satellite nodules (SN), and grading chronic liver diseases using the Batts and Ludwig scoring system (G/S stage). Additionally, reports should include HCC tissue type (Trabecular, Macro Trabecular, Pseudoadenoid and etc.), cell type (Hepatic, Clear cell, Chromophobe and etc.), and tumor capsule characteristics. Furthermore, molecular pathologic findings associated with the clonal origin of HCC, biological behavior evaluation, and prognosis/treatment-related markers should be included in clinical reference [7, 8]. In the pathological diagnosis report, most immunohistochemistry items were used to classify the histopathological type. Occasionally, literature has demonstrated that high cell proliferation index KI-67 [9], mutation of tumor suppressor gene P53 [10] and other factors are correlated with poor prognosis. In addition, incomplete tumor capsule [11], MVI [12, 13], envelope invasion (EI) [14], vascular invasion (VI) and SN [15, 16] often represent poor prognosis. However, the thickness of hepatic plates (HP), vessels encapsulating tumor clusters (VETC) [17] and some other novel biomarkers, which were recently found to correlate with prognosis and/or treatment, were seldomly included in the pathological diagnosis report.

VETC, distinct from microvascular/vascular invasion, is recognized as a predictor of micro metastases following HCC surgery [18]. VETC involves the wrapping of HCC cell clusters by tumor vascular endothelial cells, forming a cobweb-like network as visualized by anti-CD34 antibody staining [19]. Previous studies suggest that cancer cell clusters enter the circulatory system through VETC, travel to target organs via the bloodstream, and proliferate to establish new metastatic lesions [20]. The presence of VETC correlates with early recurrence, shorter overall survival (OS), and worse disease-free survival (DFS) [21]. Additionally, Mori et al. have proposed VETC as a prognostic biomarker for mortality in patients undergoing liver transplantation for HCC [22]. Combining MVI with VETC may improve the prediction of prognosis in HCC patients [23]. Furthermore, clinicopathological parameters such as tumor node metastasis (TNM) stage [24], tumor size (TS) [25], and VI [26], are known to influence prognosis. However, the potential of combining VETC with clinicopathological parameters for enhanced prognostic prediction remains unclear. Notably, a study by Fang et al. reported that patients with VETC-negative HCC treated with sorafenib had a worse prognosis than those with VETC-positive HCC [27], highlighting the need for further investigate VETC with prognosis and treatment.

In this study, our objective was to develop a prognostic model by integrating clinical and pathological parameters. We collected more than 1200 samples to establish a multi-parametric prognostic model, focusing primarily on VETC and HP, termed the VETC-HP model, to predict OS in HCC patients. Additionally, we gathered data from 37 patients treated with lenvatinib to examine the relationship between our VETC-HP model and lenvatinib treatment.

## Materials and methods

### Patient selection and data collection

A total of 1255 patients diagnosed with primary HCC from December 2000 to May 2017 and undergoing liver resection surgery at the Sun Yat-sen University Cancer Center (SYSUCC) were included in this study. None of these patients had received radiotherapy or chemotherapy prior to hepatectomy. Additionally, 37 patients treated with lenvatinib were included to explore the correlation of VETC with lenvatinib. Clinical and pathological data were collected (supplementary Table S1). The inclusion criteria were as follows: 1. Clinical and Pathological diagnosis of primary HCC; 2. Patients who underwent hepatectomy; 3. Absence of radiotherapy or chemotherapy prior to hepatectomy; 4. Absence of other malignancies prior to hepatectomy. Patients who did not have all the required parameters were excluded.

Tissue microarrays (TMA) were constructed from archived paraffin-embedded specimens and anonymized. Classic areas for TMA cores were selected by examining whole Hematoxylin–Eosin Staining (HE) slides. Six different tumoral cores were obtained. The results of VETC (evaluated by CD34 staining) on TMA were compared with whole slides for most patients. This study was approved by the Institute of Research Medical Ethics Committee of SYSUCC.

At the time of diagnosis before any treatment, clinical and serological data were collected, including age, sex, HBV DNA copy number, serum AFP, tumor size (TS), tumor multiplicity, tumor differentiation, liver cirrhosis, vascular invasion (VI), microvascular invasion (MVI), HCC tissue type, HCC cell type, tumor capsule, lymph node metastasis (LNM), VETC (evaluated by CD34 staining), Hepatic plate (HP), tumor infiltrating lymphocytes (TILs), G/S stage (according to the Batts and Ludwig score system) [28], and TNM stage, which are related to prognosis and treatment and are recommended in different guidelines for the diagnosis and treatment of HCC [7, 8, 29–31]. These clinical parameters were collected through the electronic Information System of SYSUCC. Some of these pathological parameters were also obtained from pathological diagnostic reports in the electronic Information System, while the other pathological parameters (mainly including HP and VETC) were achieved via re-evaluating slides and TMA with two independent pathologists. Patients treated with lenvatinib + surgery were designated as the lenvatinib alone group, and those treated with Lenvatinib + transcatheter arterial chemoembolization (TACE)/ transcatheter arterial infusion (TAI) + surgery were designated as the lenvatinib combined group.

### Hematoxylin and eosin (HE) and immunohistochemistry (IHC) staining

**Fig. 1:**
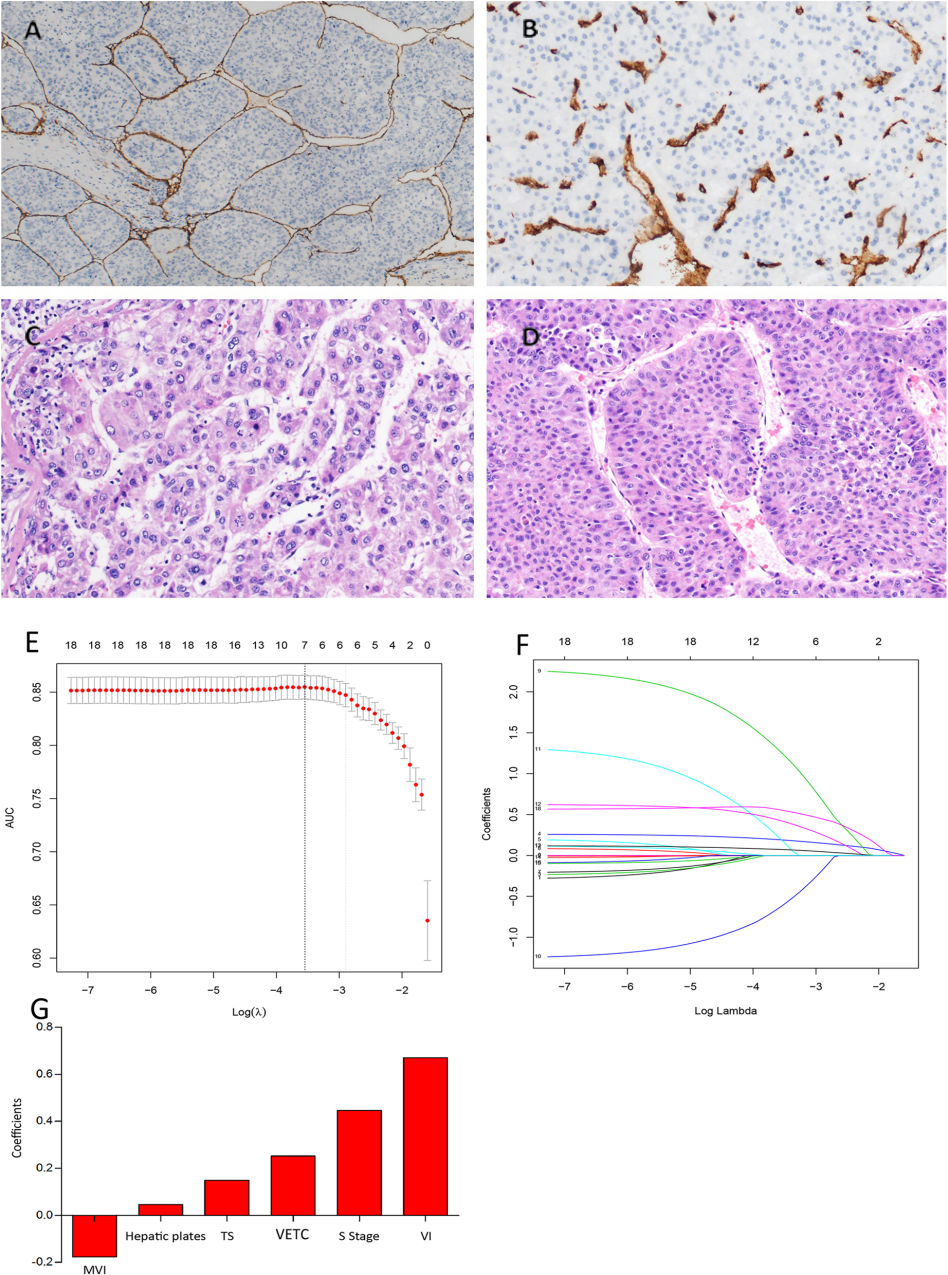
Selection of potential predictive factors using LASSO regression analysis. Representative images of **A** VETC positive, stained with CD34, **B** VETC negative, stained with CD34, **C** HP thickness < 6 layers, and **D** HP thickness ≥ 6 layers; **E** Penalty parameter in LASSO regression analysis. Tenfold cross validation and 1 standard error were used to tune the penalty parameter; **F** Changing trajectories of each predictive factor. The x-axis represents the log value of each predictive factor λ and the y-axis represents the coefficient of the independent predictive factor; **G** Histogram shows the role of each predictive factor that contributes to the constructed prognostic model. The x-axis represents the predictive factors and the y-axis represents the coefficients in the LASSO regression analysis of each predictive factor

A 4-mm slice of the TMA block was placed on a glass slide, which was dewaxed and treated with 3% hydrogen peroxide in methanol. IHC staining were performed after blocking with a biotin-blocking kit (DAKO, Hamburg, Germany). The expression pattern of CD34 (Catalog number MAB-1076, Fuzhou Maixin Biotechnology Development Company, China) in HCC was evaluated by two independent pathologists (Mei-Fang Zhang and Chun-Hua Wang). CD34-labeled vessels that encapsulated tumor clusters were identified by the presence of cobweb-like networks with unequivocal immunoreactivity of a continuous lining [17, 32]. Staining of CD34 spanning > 50% of the tumor surface was defined as VETC-positive (Fig. 1A), while staining covering ≤ 50% was deemed VETC-negative (Fig. 1B). Hepatic plates/cords < 6 cells in thickness were classified as thin plates (Fig. 1C), whereas those ≥ 6 cells in thickness were considered thick plates (Fig. 1D), and the specific cell thickness was recorded.

### Patient follow up

Follow-up data on patients were collected through medical record searches, emails, and direct telephone communication. Patients were followed up until January 2020 unless they had died. The follow-up period was defined as the time interval between the surgery and the last follow-up. OS was recorded as the time between surgery and cancer-related death or the date of the last follow-up, while DFS was defined as the time between surgery and recurrence or metastasis or the date of the last follow-up.

### Statistical analyses

Statistical analyses were conducted using IBM SPSS software (version 19.0; Chicago, IL, USA) and R version 3.6.0 (http://www.R-proje ct.org). Categorical variables were classified directly based on clinical findings, while continuous variables were transformed into categorical variables using the R packages "survival" [33] and "survminer" [34]. The distribution between patients in the training and validation sets. Was analyzed using the chi-square test. The most useful prognostic variables in the training cohort were selected via Least absolute shrinkage and selection operator (LASSO) regression analysis using the R package "glmnet" [35, 36]. Prognostic models were evaluated using Harrell’s concordance index (C-index), time-dependent receiver operating characteristics curve (tdROC), and decision curve analysis [37], as described in previous reports. The "survivalROC" package [38] and the "survcomp" package [39] were used to calculate the area under the curve (AUC) and the C-index, respectively. TNM staging and the prognostic model risk score and were used to establish a nomogram via the “rms” package [40]. For OS and DFS survival analyses, a Kaplan–Meier survival analysis and a log-rank test were performed. Pearson’s correlation coefficient [41] was employed to evaluate the association between the prognostic model, TNM staging, and lenvatinib treatment.

## Results

### Patient characteristics

A total of 1255 eligible patients were divided into a training cohort (879 cases) and a validation cohort (376 cases). The median follow-up times for OS in the training and validation cohorts were 31.7 months (interquartile range (IQR):13.0–43.1) and 32.3 months (IQR: 12.8–46.2), respectively. For DFS, the median follow-up times were 20.4 months (IQR: 6.2–38.8) and 25.5 months (IQR: 7.2–42.9) in the training and validation cohorts, respectively. In the training cohort, the OS rates were 78.5% at 1 year, 55.2% at 3 years, and 48.2% at 5 years. In the validation cohort, the corresponding rates were 77.4%, 54.3%, and 45.7%, respectively. The DFS rates at 1, 3, and 5 years in the training set were 73.4%, 62.1%, and 58.7%, respectively, and in the validation set, they were 77.4%, 68.1%, and 64.4%, respectively. Detailed clinicopathological characteristics of HCC patients and the optimal cut-off values for each continuous variable are presented in Table 1, while representative images of VETC and HP are displayed in Fig. 1A–D. The distribution of clinicopathological characteristics was similar between the training and validation cohorts.

**Table 1:** Clinicopathological characteristics of patients in the training and validation cohort

Characteristic	Training cohort (n = 879)	Validation cohort (n = 376)	*P* value
Gender			0.635
Male	758 (86.2%)	328 (87.2%)	
Female	121 (13.8%)	48 (12.8%)	
Age (years)			0.546
< 49	402 (45.7%)	165 (43.9%)	
≥ 49	477 (54.3%)	211(56.1%)	
Tumor multiplicity			0.998
Single	491 (55.9%)	210 (55.9%)	
Multiple	388 (44.1%)	166 (44.1%)	
Tumor size (cm)			0.082
< 5	319 (36.3%)	156 (41.5%)	
≥ 5	560 (63.7%)	220 (58.5%)	
HBV			0.932
Negative	149 (17.0%)	63 (16.8%)	
Positive	730 (83.0%)	313 (83.2%)	
AFP (ng/ml)			0.932
< 20	250 (28.4%)	109 (29.0%)	
≥ 20	629 (71.6%)	267 (71.0%)	
Tumor Differentiation			0.609
Well-Moderate	510 (58.0%)	224 (59.6%)	
Poor	369 (42.0%)	152 (40.4%)	
Cirrhosis			0.985
No	231 (26.3%)	99 (26.3%)	
Yes	648 (73.7%)	277 (73.7%)	
VI			0.664
No	766 (87.1%)	331 (88.0%)	
Yes	113 (12.9%)	45 (12.0%)	
MVI			0.164
No	635(72.2%)	257(68.4%)	
Yes	244(27.8%)	119(31.6%)	
Tissue type			0.955
Micro	234 (26.6%)	97 (25.8%)	
Macro	416 (47.3%)	180 (47.9%)	
Others	229 (26.1%)	99 (26.3%)	
Cell type			0.544
Hepatic	746 (84.9%)	328 (87.2%)	
Clear cell	73 (8.3%)	27 (7.2%)	
Others	60 (6.8%)	21 (5.6%)	
LNM			0.191
No	848 (96.5%)	368 (97.9%)	
Yes	31 (3.5%)	8 (2.1%)	
VETC			0.304
Negative	482 (54.8%)	218 (58.0%)	
Positive	397 (45.2%)	158 (42.0%)	
Hepatic plate			0.725
< 6	353 (40.2%)	155 (41.2%)	
≥ 6	526 (59.8%)	221 (58.8%)	
Tumor Capsule			0.869
Incomplete	414 (47.1%)	179 (47.6%)	
Complete	465 (52.9%)	197 (52.4%)	
G stage			0.934
1	17 (1.9%)	8 (2.1%)	
2	407 (46.3%)	168 (44.7%)	
3	146 (16.6%)	67 (17.8%)	
4	309 (35.2%)	133 (35.4%)	
S stage			0.727
1	17 (1.9%)	8 (2.1%)	
2	476 (54.1%)	195 (51.9%)	
3	77 (8.8%)	40 (10.6%)	
4	309 (35.2%)	133 (35.4%)	
Tils (%)			0.903
< 10	697 (79.3%)	297 (79.0%)	
≥ 10	182 (20.7%)	79 (21.0%)	
TNM^a^			0.483
I	275 (31.3%)	120 (32.2%)	
II	265 (30.1%)	126 (33.5%)	
III	279 (31.7%)	104 (27.7%)	
IV	60 (6.8%)	26 (6.9%)	

TNM tumor node metastasis stage, VI vascular invasion, MVI microvascular invasion

LNM lymph node metastasis, VETC vessels encapsulating tumor clusters, Tils tumor infiltrating lymphocytes, G stage grade of inflammation, S stage stage of fibrosis

a TNM stage was classified according to the AJCC 7th TNM staging system

### Construction of the multi-parametric prognostic model based on clinical and pathologic parameters

LASSO regression analysis was performed on the training cohort to screen for prognostic clinicopathological characteristics. The model was established using tenfold cross-validation, with an optimal λ value of 0.055. The confidence interval for each λ is depicted in Fig. 1E, while Fig. 1F illustrates the trajectory changes for each analyzed indicator. Among the 18 markers, six indicators were selected: MVI, HP, TS, VETC, S stage, and VI, as shown in Fig. 1G. Based on these clinicopathological characteristics, a multi-parametric prognostic model (VETC-HP model) was established using LASSO regression analysis. The VETC-HP model risk score was calculated using the formula: Prognostic model risk score = − 2.512 − (0.178 × MVI) + (0.046 × HP) + (0.149 × TS) + (0.252 × VETC) + (0.446 × S stage) + (0.671 × VI). In this formula, each qualitative variable is assigned a value of 0 or 1. Parameters are assigned a value of 1 for patients with positive MVI, VI, or VETC, and 0 otherwise.

### Evaluation and validation of the predictive performance of the VETC-HP model

The discriminatory performance of the VETC-HP model and TNM staging was evaluated using the C-index (Table 2 and Figure S1). In the training cohort, the C-index for OS was significantly higher for the VETC-HP model (0.753, 95% CI 0.732–0.775) compared to the TNM staging system (0.626, 95% CI 0.600–0.651, p < 0.001). Similarly, in the validation cohort, the C-index for OS was higher for the VETC-HP model (0.728, 95% CI 0.693–0.763) compared to the TNM staging system (0.629, 95% CI 0.592–0.666, p < 0.001). For DFS, the VETC-HP model also outperformed the TNM staging system in both cohorts: 0.672 (95% CI 0.646–0.698) vs. 0.573 (95% CI 0.544–0.603, p < 0.001) in the training cohort, and 0.615 (95% CI 0.564–0.666) vs. 0.511 (95% CI 0.462–0.560, p < 0.001) in the validation cohort. These results indicate that the VETC-HP model demonstrates superior discriminatory ability compared to the TNM staging system.

**Table 2:** The C-index of our model, TNM stage for prediction of OS and DFS in HCC in the training cohort and validation cohort

	C-index	95 CI%	*P*
Training cohort			
For OS			
Our model	0.753	0.732–0.775	
TNM stage	0.626	0.600–0.651	
Our model vs TNM stage			< 0.001
For DFS			
Our model	0.672	0.646–0.698	
TNM stage	0.573	0.544–0.603	
Our model vs TNM stage			< 0.001
Validation cohort			
For OS			
Our model	0.728	0.693–0.763	
TNM stage	0.629	0.592–0.666	
Our model vs TNM stage			< 0.001
For DFS			
Our model	0.615	0.564–0.666	
TNM stage	0.511	0.462–0.560	
Our model vs TNM stage			< 0.001

Our model: Tumor size + Vascular Invasion + microVascular Invasion + VETC (evaluated by CD34) + Hepatic plate + S stage

C-index = concordance index; P values are calculated based on normal approximation using function rcorrp.cens in Hmisc package

**Fig. 2:**
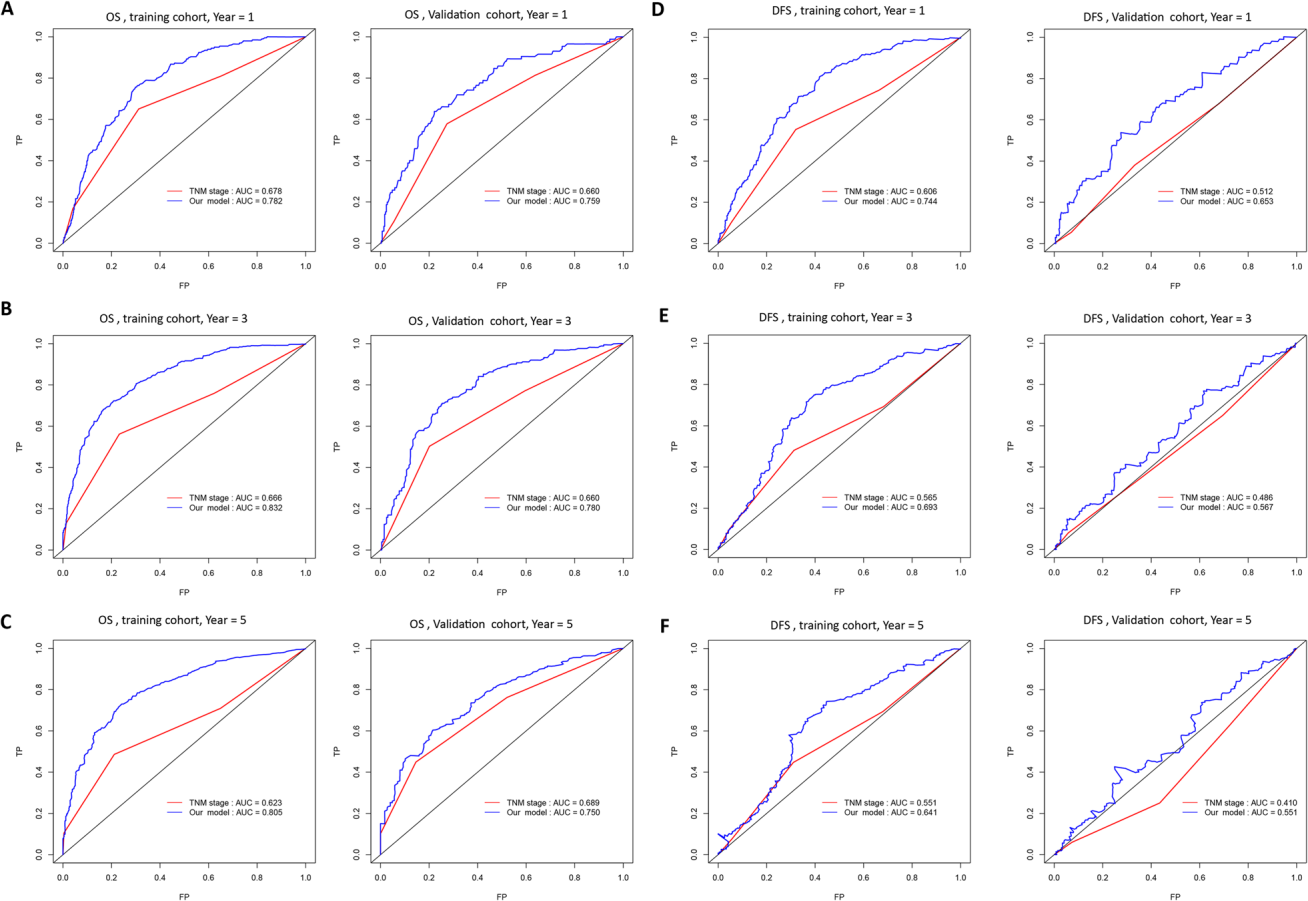
Predictive accuracy comparison between the prognostic model and TNM staging. Time dependent ROC curves at 1, 3, and 5 years **A–C** for OS, **D**–**F** for DFS in the training set (left) and the validation set (right)

The tdROC analysis further supported the superior prognostic accuracy of the VETC-HP model over TNM staging. The AUCs for 1-year, 3-year, and 5-year overall survival in the training set were 0.782, 0.832, and 0.805, respectively, for the VETC-HP model, compared to lower AUCs for TNM staging (0.678, 0.666, and 0.623, respectively). Similarly, in the validation cohort, the VETC-HP model exhibited higher AUCs for both OS and DFS compared to the TNM staging system (Fig. 2). These results consistently indicate that the VETC-HP model provides better predictions of survival time than the TNM staging system.

**Fig. 3:**
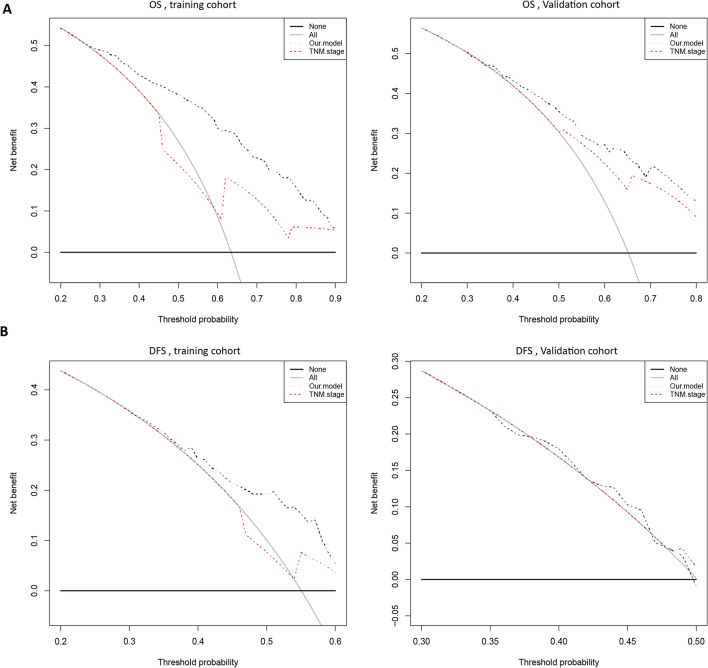
Decision curve analysis for each model. OS (**A**) and DFS (**B**) in the training set (left) and the validation set (right). The black line represents the net benefit for our prognostic model, and the red line represents the net benefit of TNM stage. The y-axis represents the net benefit and the x-axis represents the threshold probabilities

Decision curve analysis demonstrated that the VETC-HP model for OS and DFS had a superior overall net benefit compared to the TNM staging system across a wide range of threshold probabilities in both the training and validation sets (Fig. 3).

### Establishment of a nomogram based on prognostic model risk score

**Fig. 4:**
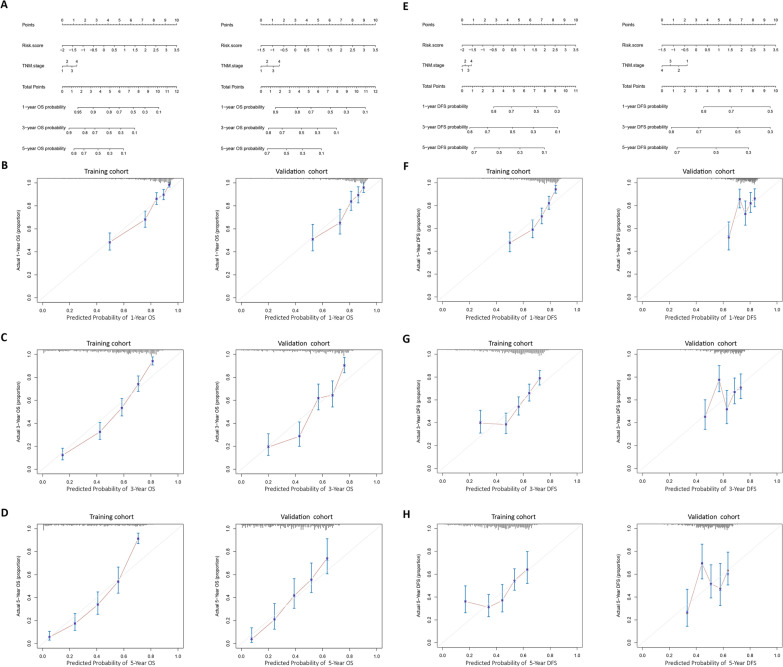
Nomograms to predict OS (**A**) and DFS (**E**) for HCC patients. Calibration plots (**B–D** for OS, **F–H** for DFS) for the nomograms at 1, 3, and 5 years in the training set (left) and the validation set (right). The gray line indicates the ideal reference line where predicted probabilities would match the observed survival rates. The red dots are calculated by bootstrapping and represent the performance of the nomogram

Based on the prognostic model risk score and TNM staging, we established a nomogram to predict OS and DFS at 1, 3, and 5 years (Fig. 4A and E). Each subtype was assigned a point based on its variable, and the nomogram was used to determine the probability of OS and DFS at specified time points. To utilize the nomogram for individual patients, their information for the risk score and TNM stage (represented on axes 2 and 3) should be plotted as a point on the first axis. The sum of these two points, relative to the total number of points, is then marked on axis 4. A line is then drawn downward to the risk axis (axes 5–7) to ascertain the likelihood of recurrence for that patient at 1, 3, and 5 years.

Calibration plots were used to assess the consistency between the predictions made by the nomogram and actual observations (Fig. 4B–D and F–H). These plots demonstrated good agreement between the nomogram predictions and observed probabilities of OS and DFS at 1, 3, and 5 years.

### Performance of VETC-HP model risk score in risk stratification

The distribution of risk scores and the optimal cutoff value (0.05) for the VETC-HP model are illustrated in Figure S2. Patients with a risk score ≤ 0.05 were classified as low-risk, while those with a score > 0.05 were classified as high-risk. Table 3 presents the OS and DFS rates for the high- and low-risk groups in both the training and validation cohorts.

**Table 3:** OS and DFS rate in high-risk and low-risk groups according to our model risk score in the training and validation cohort

Parameter	training cohort			Validation cohort		
	High-risk group	Low-risk group	Total	High-risk group	Low-risk group	Total
No. of patients	471	408	879	195	181	376
OS						
Median	18.5	38.4		20.4	38.2	
IQR	8.0–36.8	30.6–45.4		7.8–42.4	29.7–46.8	
No. of OS						
At 1 year	308 (65.4%)	382 (93.6%)	690 (78.5%)	128 (65.6%)	163 (90.1%)	291 (77.4%)
At 3 year	153 (32.5%)	332 (81.4%)	485 (55.2%)	66 (33.8%)	138 (76.2%)	204 (54.3%)
At 5 year	108 (22.9%)	316 (77.5%)	424 (48.2%)	48 (24.6%)	124 (68.5%)	172 (45.7%)
DFS						
Median	8.4	35.75		11.9	34.3	
IQR	3.6–24.6	19.6–43.2		45–36.2	14.9–44.0	
No. of DFS						
At 1 year	289 (61.4%)	356 (87.3%)	645 (73.4%)	138 (70.8%)	153 (84.5%)	291 (77.4%)
At 3 year	244 (51.8%)	302 (74.0%)	546 (62.1%)	130 (66.7%)	126 (69.6%)	256 (68.1%)
At 5 year	233 (49.5%)	283 (69.4%)	516 (58.7%)	124 (63.6%)	118 (65.2%)	242 (64.4%)

OS overall survival, DFS disease free survival, IQR interquartile range

**Fig. 5:**
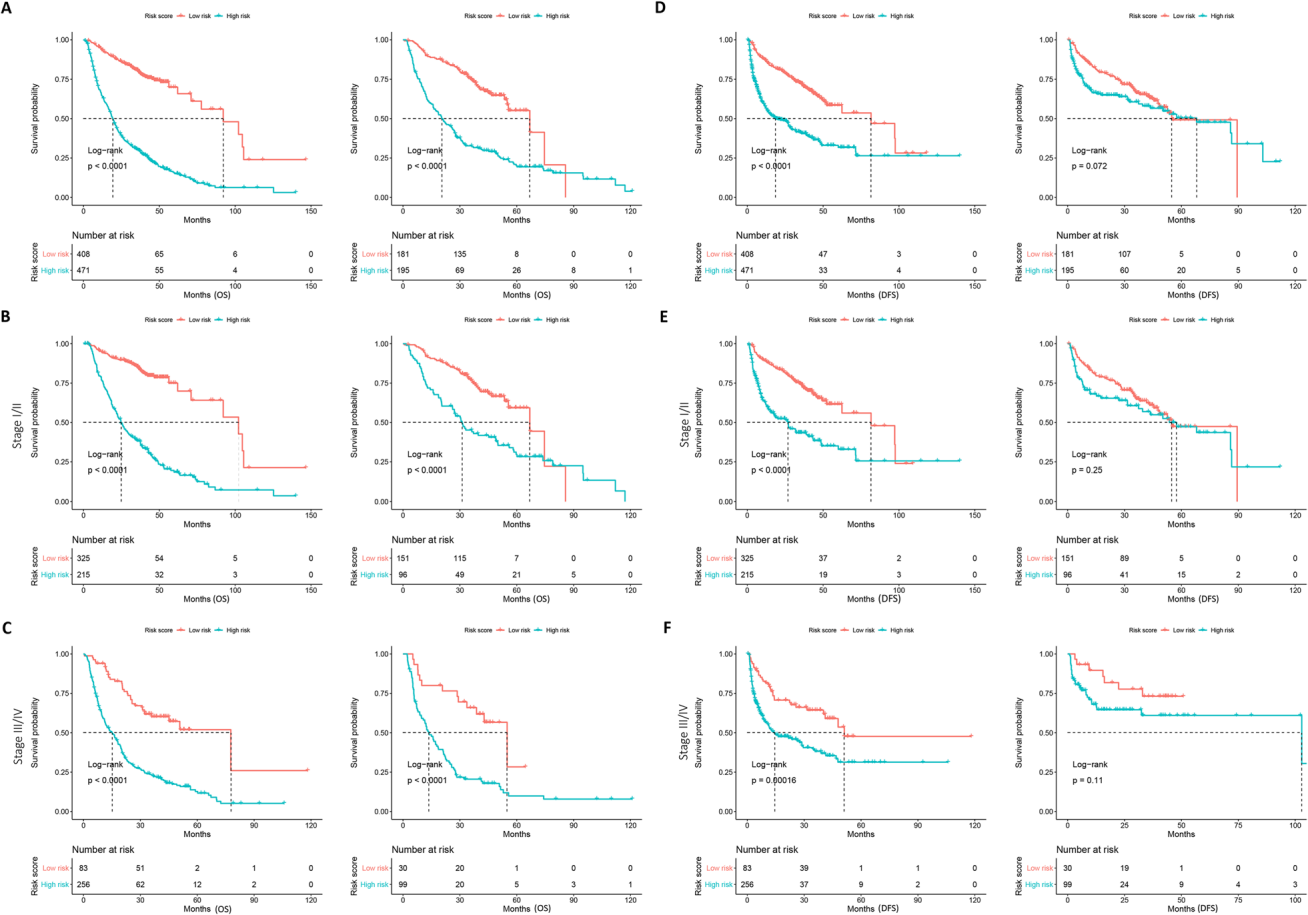
Kaplan–Meier analyses of OS and DFS according to the prognostic model risk score classifier in subgroups of HCC patients in the training set (left) and the validation set (right): total patients (**A** for OS, **D** for DFS); stage I/II (**B** for OS, **E** for DFS); stage III/IV (**C** for OS, **F** for DFS)

In the training cohort, the high-risk group had a median OS of 18.5 months (IQR: 8.0–36.8 months), compared to 38.4 months (IQR: 30.6–45.4 months) for the low-risk group. The survival probabilities at 1, 3, and 5 years for the high-risk group were 65.4%, 32.5%, and 22.9%, respectively. In contrast, the low-risk group had higher survival probabilities at 1, 3, and 5 years, with rates of 93.6%, 81.4%, and 77.5%, respectively (Table 3). Kaplan–Meier analysis of OS revealed significantly worse outcomes for patients in the high-risk group in both the training and validation cohorts (Fig. 5A). This trend was consistent across stage I/II and III/IV subgroups (Fig. 5B, C).

For DFS, the high-risk group in the training cohort had a median DFS of 8.4 months (IQR: 3.2–24.6 months), while the low-risk group had a median DFS of 35.75 months (IQR: 19.6–43.2 months). The DFS probabilities at 1, 3, and 5 years for the high-risk group were 61.4%, 51.8%, and 49.5%, respectively, compared to 87.3%, 74.0%, and 69.4%, respectively, for the low-risk group (Table 3). Kaplan–Meier analysis of DFS indicated worse prognoses for the high-risk group in the training cohort, although no significant difference was observed in the validation cohort (Fig. 5D). Stratified analysis by stage status showed consistent results, with the low-risk group demonstrating better DFS prognoses in both stage I/II and stage III/IV in the training cohort, whereas the validation cohort did not show a significant difference in DFS between the two groups (Fig. 5E, F).

### Correlation of VETC-HP prognostic model with six factors and Lenvatinib treatment

The distributions of the six predictors contributing to our prognostic model are depicted for the training and validation cohorts in Figure S3. Factors such as MVI (Figure S3A), S stage (Figure S3E), and VI (Figure S3F) did not significantly differ between the high-risk and low-risk groups. However, VETC (Figure S3D), HP (Figure S3B), and TS (Figure S3C) showed marked differences. The high-risk group exhibited a higher prevalence of VETC-positive patients (Figure S3D), thicker HP (Figure S3B), and larger tumor sizes (Figure S3C) compared to the low-risk group.

**Fig. 6:**
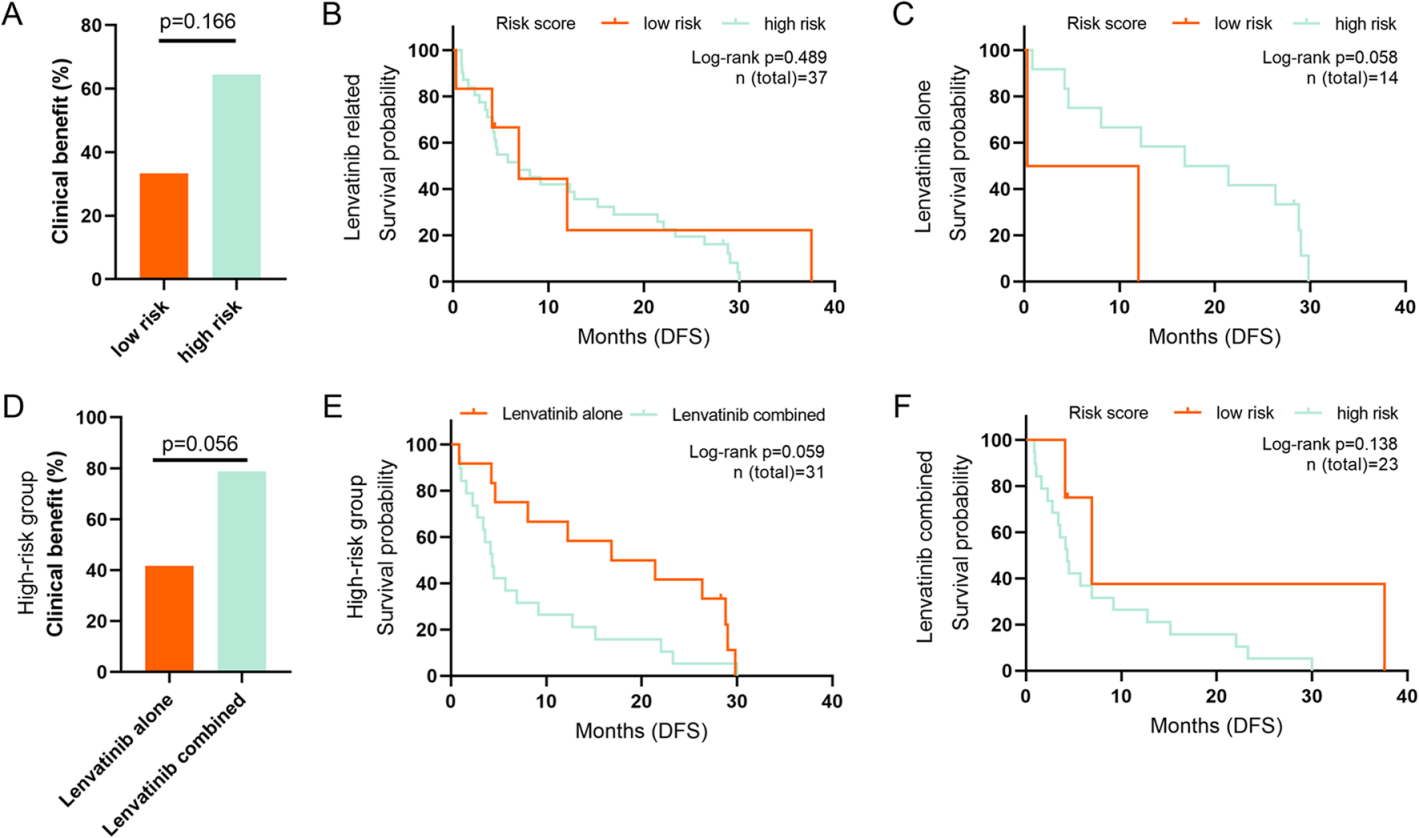
Clinical benefit ratio between the high-risk group and the low-risk group in Lenvatinib treated patients (**A**), and between the Lenvatinib alone group and the Lenvatinib combined group in the high-risk group (**D**). Kaplan–Meier analyses of DFS between the high-risk group and the low-risk group in all Lenvatinib-treated patients (**B**), Lenvatinib alone patients, Lenvatinib + surgery group (**C**), and Lenvatinib combined patients, Lenvatinib + TACE/TAI + surgery group (**F**). Kaplan–Meier analyses of DFS between the Lenvatinib alone group and the Lenvatinib combined group in the high-risk group (**E**)

Previously, a study indicated a correlation between the VETC pattern and the efficacy of sorafenib treatment in HCC patients [27]. And our results showed that VETC positive patients were more in high-risk group than in low-risk group. Thus, we included another 37 patients treated with lenvatinib. There were no significant differences between the high- and low-risk groups in terms of clinical benefit (Fig. 6A) or DFS prognosis (Fig. 6B). However, in the Lenvatinib + surgery group (lenvatinib alone), the high-risk group showed a trend towards better DFS prognosis (log-rank p = 0.058, Fig. 6C), while no difference was observed in the lenvatinib + TACE/TAI + surgery group (lenvatinib combined) (Fig. 6F). Furthermore, in the high-risk group, the lenvatinib combined group exhibited a slightly, but not significantly, higher clinical benefit than the lenvatinib alone group (p = 0.056, Fig. 6D), while the lenvatinib alone group had a better DFS prognosis (log-rank p = 0.059, Fig. 6E).

## Discussion

In this study, we used VETC combined with other clinical pathological parameters to establish a multi-parametric prognostic model for HCC prognosis prediction. Initially, the VETC-HP model, capable of predicting survival rates with high accuracy, was established using the LASSO regression assay, a method employed in previous studies [36]. This model represents an innovative prognostic tool that outperforms the TNM staging system in predicting survival outcomes for HCC patients, providing valuable clinical research insights into incorporating VETC and HP characteristics into pathology reports.

In the present guidelines for the pathological diagnosis of HCC, gross description of specimens, microscopic description, pathological diagnosis, and immunohistochemical examination results are required. Additionally, molecular pathologic findings, biological behavior evaluations, and prognosis/treatment-related markers should be included in clinical reference [7, 8]. However, most pathological reports do not include the prognostically significant VETC and hepatic plate thickness. Our study underscores the importance of including these parameters in HCC pathology reports based on the findings of our novel VETC-HP model, which demonstrates their significance in prognosis and treatment planning.

While prior studies have indicated a correlation between VETC and the prognosis of HCC patients [23, 42, 43], our study did not find VETC to be an independent prognostic indicator in a cohort of 1255 patients. Nonetheless, recognizing the potential prognostic value of VETC, we sought to develop a highly efficient prognostic model centered on VETC. Consequently, we constructed the VETC-HP model, which combines VETC with other clinicopathological parameters. The prognostic performance of the VETC-HP model surpassed that of the conventional TNM staging system. This study, one of the largest retrospective research endeavors, has led to the development of an effective prognostic model for HCC patients. Notably, the VETC-HP model includes only six easily evaluated pathological characteristics, making it a simple and convenient tool for predicting HCC prognosis.

Our VETC-HP, based on 18 parameters, identified 6 significant hazard ratios. This model was developed using a training cohort and validated with a separate cohort. Our results demonstrated a clear distinction in OS curves between patients with high and low scores. Notably, the VETC-HP model outperformed the current TNM staging system in predicting OS. These results suggest that the VETC-HP model could serve as a valuable tool for prognosis prediction, complementing the existing TNM staging system. Additionally, the nomogram, incorporating the VETC-HP model and TNM stage, exhibited superior prognostic value compared to TNM staging alone, enhancing the predictive strength of the traditional TNM method. These results were consistent in the validation cohort, confirming the utility of the VETC-HP model in HCC and potentially advancing.

To date, only one study has investigated the relationship between VETC and sorafenib treatment [27]. Given that lenvatinib shares a similar mechanism of action with sorafenib, we explored the association between the VETC-HP model and lenvatinib treatment. Although no significant differences were observed in clinical benefit, DFS, and other parameters, likely due to the small sample size, some trends were noted. Specifically, in the high-risk group, the lenvatinib combined group showed a slightly higher clinical benefit compared to the lenvatinib alone group (p = 0.056), while the lenvatinib combined group may have had a poorer DFS prognosis than the lenvatinib alone group (log-rank p = 0.059). Moreover, in the lenvatinib alone group, the high-risk group might have exhibited a better DFS prognosis than the low-risk group. And in our study, the high-risk group had more VETC-positive patients than VETC-negative patients. The trends of our results were similar to Fang and his colleagues’ study, which was found that patients with VETC-negative HCC treated with sorafenib had a worse prognosis than those with VETC-positive HCC [27]. Further, Zhang and his colleagues found that gene expression levels of fibroblast growth factor receptors were upregulated in VETC-positive HCC, which suggested that VETC-positive HCC might benefit from Lenvatinib treatment [32]. Therefore, previous report and our study imply that high-risk VETC-HP patients may be more suitable for treatment with lenvatinib alone, thus providing clinical evidence for the use of lenvatinib in personalized clinical treatment.

Given the retrospective nature of this study, the limitations in reliability on data collection and selection bias should be noted. Specifically, we were unable to retrospectively collect data on the protein level induced by vitamin K absence or antagonist-II (PIVIKA II), a newly discovered serological marker of HCC, for all patients; therefore, this parameter was excluded from our analysis. Additionally, some patients transferred to other hospitals post-surgery, making it difficult to track the trajectory of adjunct therapy following surgery. Moreover, due to our sample size, further research is needed to fully elucidate the relationship between lenvatinib treatment and our model.

### Supplementary Information

Supplementary Material 1: Figure S1. C-index comparison between the prognostic model and TNM staging for OS (A) and DFS (B) in the training set (left) and the validation set (right). Figure S2. Distribution of risk scores (upper panel) and the determination of optimal cut-off value (lower panel). Figure S3. Distribution of the six predictors that contributing to the developed prognostic model in the training set (left) and the validation set (right): MVI (**A**), HP (**B**), Tumor size (**C**), VETC (**D**), S stage (**E**), VI (**F**).

## Data Availability

The authenticity of this article was validated by uploading the key raw data onto the Research Data Deposit public platform (www.researchdata.org.cn) with the approval RDD number RDDB2023291512. All data included in this study are available upon request from the corresponding author.

## Abbreviations

VETC: Vessels encapsulating tumor clusters
HP: Hepatic plates
LASSO: Least absolute shrinkage and selection operator
HCC: Hepatocellular carcinoma
MVI: Microvascular invasion
TNM: Tumor node metastasis
OS: Overall survival
AUC: Area under the curve
DFS: Disease-free survival
TS: Tumor size
VI: Vascular invasion
GS score system: G stage and S stage, Batts and Ludwig score system
