# Supplementary material for: A multi-parametric prognostic model based on clinicopathologic features: vessels encapsulating tumor clusters and hepatic plates predict overall survival in hepatocellular carcinoma patients
Source: J Transl Med. 2024 May 18;22:472. doi: 10.1186/s12967-024-05296-3 (PMC11102615; doi:10.1186/s12967-024-05296-3)
Supplement: Supplementary file 1 — Supplementary Material 1: Figure S1. C-index comparison between the prognostic model and TNM staging for OS (A) and DFS (B) in the training set (left) and the validation set (right). Figure S2. Distribution of risk scores (upper panel) and the determination of optimal cut-off value (lower panel). Figure S3. Distribution of the six predictors that contributing to the developed prognostic model in the training set (left) and the validation set (right): MVI (A), HP (B), Tumor size (C), VETC (D), S stage (E), VI (F). [file 12967_2024_5296_MOESM1_ESM.docx]

**Table S1. Patient demographics and clinical characteristics**

| **Characteristic** | **n=1255** | **n=37(Lenvatib alone,n=14;**  **Lenvatib combined,n=23)** |
| --- | --- | --- |
| **Gender** |  |  |
| Male | 1086(86.5%) | 34(91.9%) |
| Female | 169(13.5%) | 3(8.1%) |
| **Median age (years)** | 50 (13-84) | 52(22-67) |
| **Average size of Tumor（cm）** | 6.7 (0.7-24) | 9.2(3-16.7) |
| **HBV** |  |  |
| Negative | 212(16.9%) | 34(91.9%) |
| Positive | 1043(83.1%) | 3(8.1%) |
| **AFP** |  |  |
| ＜ 20 ng/ml | 359(28.6%) | 6(16.2%) |
| ≥ 20 ng/ml | 896(71.4%) | 31(83.8%) |
| **TNM^a^** |  |  |
| I | 395(31.5%) | 3(8.1%) |
| II | 391(31.2%) | 7(18.9%) |
| III | 383(30.5%) | 12(32.5%) |
| IV | 86(6.8%) | 15(40.5%) |
| **LNM** |  |  |
| No | 1216(96.9%) | 31(83.8%) |
| Yes | 39(3.1%) | 6(16.2%) |
| **Tumor Differentiation** |  |  |
| Well-Moderate | 734(58.5%) | 11(29.7%) |
| Poor | 521(41.5%) | 26(70.3%) |
| **Cirrhosis** |  |  |
| No | 330(26.3%) | 16(43.2%) |
| Yes | 925(73.7%) | 21(56.8%) |
| **Vascular invasion** |  |  |
| No | 1097(87.4%) | 12(32.5%) |
| Yes | 158(12.6%) | 25(67.5%) |
| **Microvascular Invasion** |  |  |
| No | 892(71.1%) | 27(73.0%) |
| Yes | 363(28.9%) | 10(27.0%) |
| **VETC(CD34)** |  |  |
| No | 700(55.8%) | 25(67.5%) |
| Yes | 555(44.2%) | 12(32.5%) |
| **Hepatic Plate** |  |  |
| ＜ 6 | 508(86.5%) | 13(35.1%) |
| ≥ 6 | 747(40.5%) | 24(64.9%) |
| **G stage^b^** |  |  |
| 1-2 | 600(47.8%) | 22(59.5%) |
| 3-4 | 655(52.2%) | 15(40.5%) |
| **S stage^b^** |  |  |
| 1-2 | 696(55.5%) | 21(56.8%) |
| 3-4 | 560(44.5%) | 16(43.2%) |
| **Stromal TILs** |  |  |
| ＜ 10 | 994(79.2%) | 30(81.1%) |
| ≥ 10 | 261(20.8%) | 7(18.9%) |
| **Median DFS (months)** | 21.7(0.4-140) | 6.9(0.3-37.6) |
| **Median OS (months)** | 31.8(0.4-146.6) | 16.9(0.3-60.2) |

TNM tumor node metastasis stage, LNM lymph node metastasis, Tils tumor infiltrating lymphocytes, G stage grade of inflammation, S stage of fibrosis.

Lenvatib alone: treatment with Lenvatib and surgery.

Lenvatib combined: treatment with Lenvatib, TACE/TAI and surgery.

a TNM stage was classified according to the AJCC 8th TNM staging system.

b GS stage was classified according to Batts and Ludwig score system


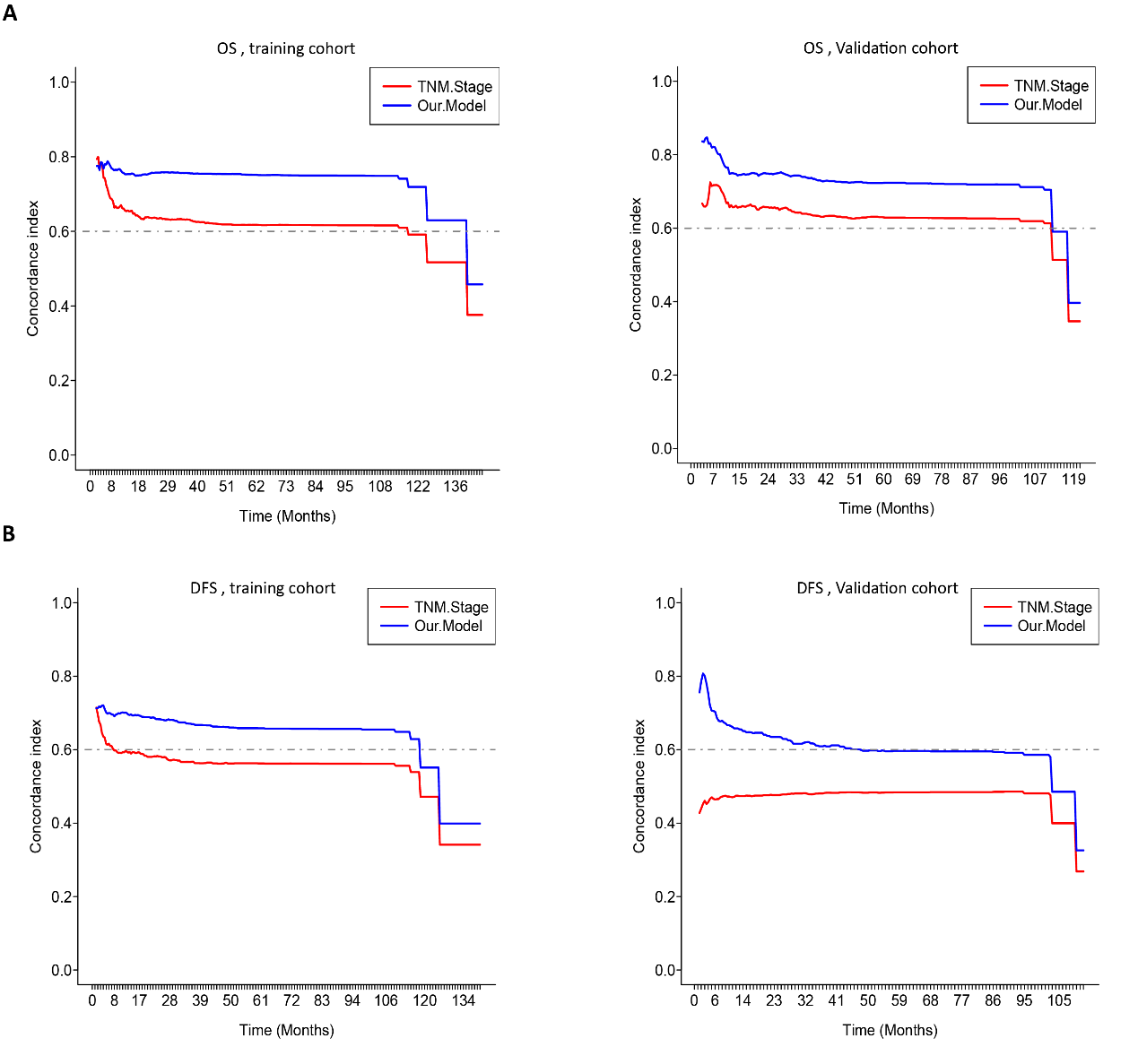


Supplementary Figure S1. C-index comparison between the prognostic model and TNM staging for OS (A) and DFS (B) in the training set (left) and the validation set (right).


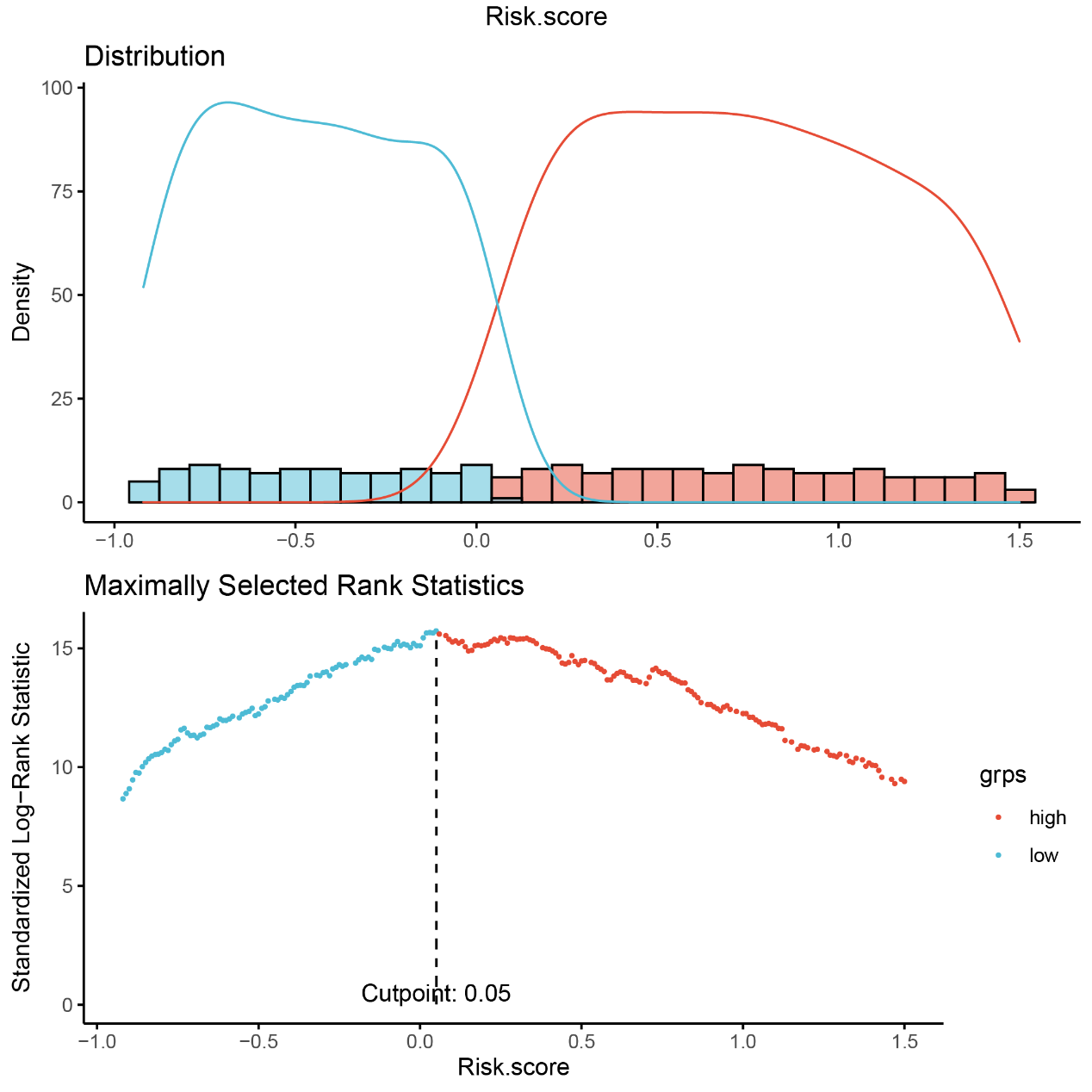


Supplementary Figure S2. Distribution of risk scores (upper panel) and the determination of optimal cut-off value (lower panel).


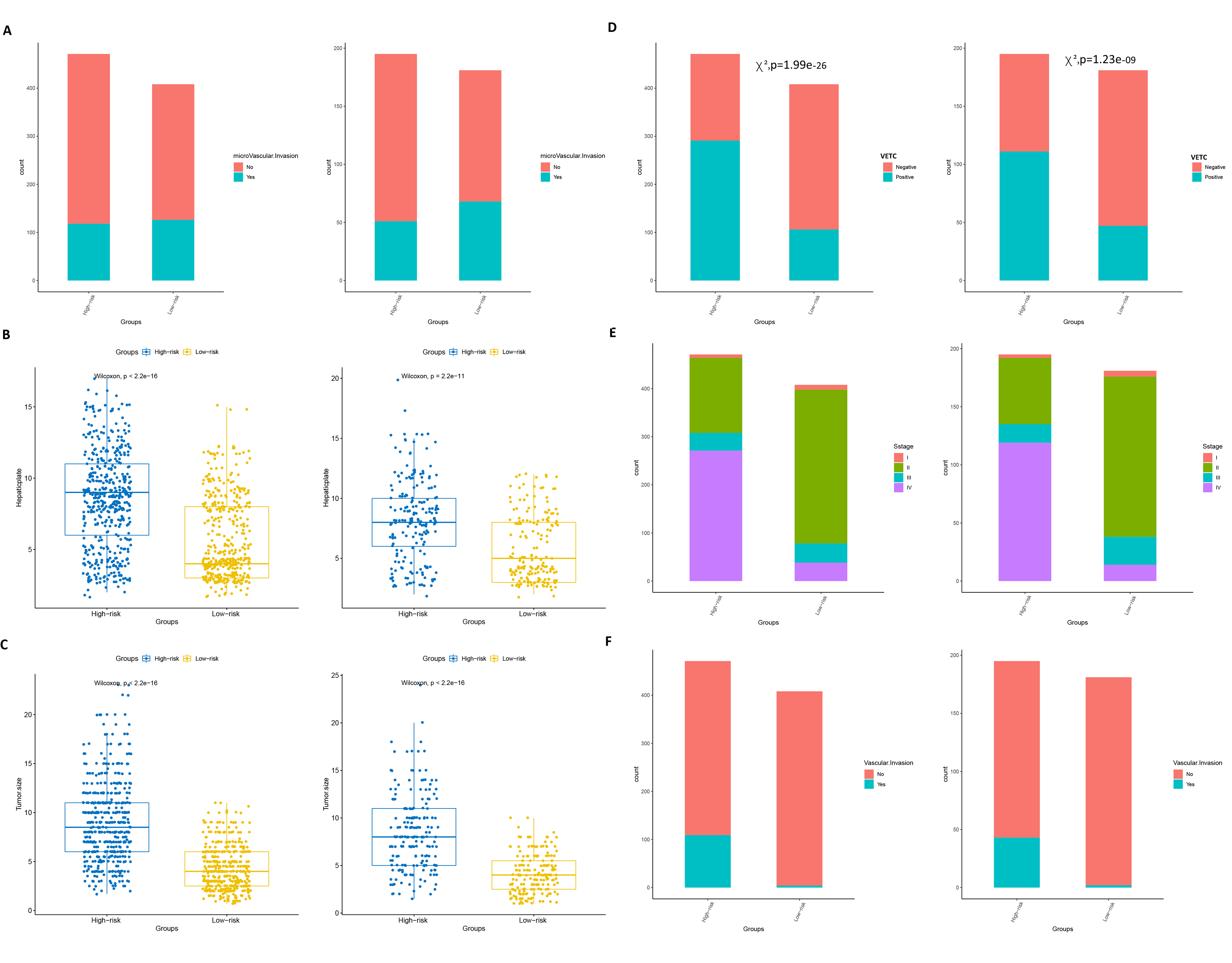


Supplementary Figure S3. Distribution of the six predictors that contributing to the developed prognostic model in the training set (left) and the validation set (right): MVI (A), HP (B), Tumor size (C), VETC (D), S stage (E), VI (F).
